# Serum Vitamin D and Vitamin D Receptor Gene Polymorphism in Mycosis Fungoides Patients: A Case Control Study

**DOI:** 10.1371/journal.pone.0158014

**Published:** 2016-06-23

**Authors:** Hoda Rasheed, Rehab A. Hegazy, Heba I. Gawdat, Dina A. Mehaney, Marwa M. Kamel, Marwa M. Fawzy, Mohammed M. Nooh, Hebatallah A. Darwish

**Affiliations:** 1 Dermatology Department, Faculty of Medicine, Cairo University, Cairo, Egypt; 2 Clinical and Chemical Pathology Department, Faculty of Medicine, Cairo University, Cairo, Egypt; 3 Biochemistry Department, Faculty of Pharmacy, Cairo University, Cairo, Egypt; Medical University of Gdańsk, POLAND

## Abstract

**Background:**

Vitamin D has been considered a key player in various malignancies including cutaneous cancers. To date, mycosis fungoides (MF) has been the least studied in relation to vitamin D. Furthermore, the vitamin D receptor (*VDR)* single nucleotide polymorphisms (SNPs) have not been tackled before in the context of MF, despite their incrimination in numerous diseases.

**Aim of study:**

To assess the role of vitamin D in MF by measuring its serum level, and studying VDR SNPs (*Taq*I, *Bsm*I, *Fok*I) in different stages of MF.

**Patients and Methods:**

48 patients with various stages of MF, and 45 healthy controls were included. Complete history, full clinical examination and a five mm punch skin biopsy were performed to all recruited patients. Venous blood samples were withdrawn from both patients and controls to determine the serum vitamin D level and VDR gene polymorphisms.

**Results:**

Serum vitamin D level was significantly lower in patients (5.3–33.7 nmol/L)] compared to controls (8.3–90.1 nmol/L)] (*P*<0.001). A significant difference was observed between patients and controls regarding the *Fok*I polymorphism only, being higher in patients (*P* = 0.039). Also Vitamin D serum levels differed significantly in patients with *Fok*I genotypes (*P* = 0.014). No significant correlations were detected between any of the studied parameters and the demographic and clinical data of the included subjects.

**Conclusion:**

Depressed vitamin D and *Fok*I polymorphism are potentially involved in the context of MF. VDR gene polymorphisms warrant further larger scale investigations to detect the exact genes involved in the pathogenesis of such an enigmatic disease.

## Introduction

Vitamin D passes through a tandem activation process. It is initially converted to 25 (OH) D3 which is the most reliable measure of vitamin D status in the human body. This is followed by its subsequent conversion to the active form 1, 25(OH) 2 D3 (calcitriol) [1].

The active form of vitamin D [1, 25 (OH) 2D3] is involved in the regulation of various metabolic processes such as cellular proliferation and differentiation through binding to vitamin D receptor (*VDR*). The role played by vitamin D in these vital processes led to the inference of it being an inhibitor of carcinogenesis [2]. Moreover, vitamin D deficiency has been associated with both the occurrence and gravity of a variety of cancers [3]. There is paucity in the literature addressing the involvement of vitamin D in cutaneous cancers, with controversial reports explaining their complex relationship [4–6].

Mycosis fungoides (MF), a low-grade lympho-proliferative disorder, is the most common type of cutaneous T cell lymphoma (CTCL). The standard treatment for early stage MF is phototherapy [7] and several previous studies demonstrated the efficacy of 1, 25(OH) 2 D3 in the treatment of CTCL [8–11]. This raises the speculation around the possible role played by vitamin D in this group of cutaneous tumors with their unique behavior.

To date, among the different cutaneous malignancies, MF has been the least studied in relation to vitamin D. There is reported evidence on the *VDR* single nucleotide polymorphisms (SNPs) that points out four SNPs with possible disease association; three at the 3' end of the *VDR* gene (*Taq*I, *Bsm*I, *Apa*I) and one at the 5’ end (*Fok*I) [12]. These SNPs have not been studied before in the context of MF. This was the impetus behind the current work, in a trial to investigate whether a relationship exists between vitamin D and MF, through measuring its serum level and studying the VDR gene polymorphism (*Taq*I, *Bsm*I, *Fok*I) in different stages of MF.

## Materials and Methods

This case control study was approved by the Dermatology Research Ethical Committee (DREC), and in accordance with the Declaration of Helsinki principles. The patients and controls were recruited from the Cairo University Hospital, Dermatology Outpatient clinic. Written informed consents were retrieved from all participants.

The study included 48 patients with various stages of MF. Forty five age and sex matched healthy controls with no known dermatological and/or systemic diseases were also included. Since this is a vitamin D research, extremes of age, accommodation in high altitudes, pregnancy, lactation, presence of comorbidities in MF patients, and intake of vitamin D dietary supplements precluded inclusion in the study [13, 14]. Furthermore, all included subjects had a skin phototype of SPT III-V.

The whole study was carried out over 4 months (July–October) with nearly the same climate parameters (all summer months; an Egyptian summer is defined as May–October by many sites). The study was performed on patients and controls from one clinic (Dermatology Outpatient Clinic, Cairo University), serving subjects with the same socioeconomic status, practices and culture.

Each patient was subjected to complete history taking, full clinical examination to detect the extent (using rule of nines) and type of MF. The diagnosis was confirmed by a histopathological examination of a 5mm punch skin biopsy taken from the suspected lesion and stained by haematoxylin and eosin stain (H & E). All biopsies were examined by a single qualified dermatopathologist.

Staging of MF according to the European Organization of Research and Treatment of Cancer (EORTC) [15] was further completed by a lymph node examination and biopsy (when indicated) in the surgery outpatient clinic, Faculty of Medicine, Cairo University. Laboratory investigations (β_2_microglobulin and lactate dehydrogenase) and radiological investigations (chest X-ray and abdominal ultrasound) were then carried out.

All included patients were refrained from any systemic or topical treatment apart from emollients for a period of four weeks before blood samples were taken.

### Assessment of Vitamin D

#### Laboratory investigations to assess vitamin D status

Venous blood samples were collected from both cases and controls and the serum was separated by centrifugation and stored at -20 °C. Serum concentrations of 25 (OH) D3 and 1, 25 (OH) 2 D3 were used as an index of vitamin D status and were determined using a competitive protein binding radioimmunoassay (25(OH) D3 125 RIA kit; DiaSorin, MN, USA). 25(OH) D3 levels were categorized as deficient (<50 nmol/L), insufficient (<75 nmol/L), and sufficient (>75 nmol/L) according to recent guidelines [16].

### Molecular analysis of VDR polymorphisms

#### DNA extraction

Five ml of venous blood was withdrawn into an EDTA tube. DNA was extracted from the peripheral blood leucocytes using the standard salting out technique [17].

#### Genotyping

Amplification and Restriction Fragment Length Polymorphism (RFLP) analysis of *Fok*I, *Taq*I and *Bsm*I VDR polymorphisms were done according to *Bid et al*., *2009* [18]. The PCR reactions were as follows: 10X buffer without MgCl_2_, 25 mMdNTPs, 5U/ul Dream *Taq* polymerase (MBI Fermentas, Vilnius, Lithuania), 100 ng DNA, and 0.4 mM of each primer.

The *Fok*IT/C SNP (rs2228570) was investigated using the following primers: Forward 5'- AGC TGG CCC TGG CAC TGA CTC TGC TCT-3', Reverse-5'- ATG GAA ACA CCT TGC TTC TTC TCC CTC-3'. The *Bsm*I A/G SNP (rs1544410) was investigated using the following primers: Forward 5'- CAA CCA AGA CTA CAA GTA CCG CGT CAG TGA-3', Reverse-5'- AAC CAG CGG GAA GAG GTC AAG GG-3'. The *Taq*I C/T SNP (rs1544410) was tested using the following primers: Forward 5'- CAG AGC ATG GAC AGG GAG CAA-3', Reverse-5'- GCA ACT CCT CAT GGC TGA GGT CTC-3'. PCR reactions were done using the PCR express thermal cycler (Thermo Hybaid, Middlesex, UK).

The PCR conditions were as follows: initial denaturation at 94°C for 10 minutes (1 cycle), denaturation at 94°C for 30seconds, annealing at 58, 63 and 63°C (for *Fok*I, *Taq*I and *Bsm*I respectively) for 30 seconds and extension at 72°C for 30 seconds (35 cycles), with a final extension at 72°C for 10 minutes.

To identify the genotypes of *Fok*I, *Bsm*I *and Taq*I polymorphisms, the PCR products were digested by the corresponding restriction enzyme (MBI Fermentas, Vilnius, Lithuania). The digestion fragments were separated by 15% polyacrylamide gel using the BioRad Mini-Protean tetra gel system (Bio-Rad, Hercules, CA, USA) and stained with ethidium bromide. A 100 bp ladder (MBI Fermentas) was used as a size marker.

For the *Fok*I polymorphism, the TT genotype produced 196 and 69 bp fragments, the CC genotype produced 265 bp fragments and the TC genotype produced 265, 196, 69 bp fragments. For the *Bsm*I polymorphism, the AA genotype produced 825 bp fragments, the GG genotype produced 650 and 175 bp fragments and the GA genotype produced 825, 650, 175 bp fragments. For the *Taq*I polymorphism, the CC genotype produced 290, 245 and 205 bp fragments, the TT genotype produced 495 and 245 bp fragments and the CT genotype produced 495, 290, 245 and 205 bp fragments.

### Statistical analysis

Data were statistically described in terms of mean ± standard deviation (± SD), median and range, or frequencies (number of cases) and percentages when appropriate. Comparison of numerical variables between the study groups was done using Mann Whitney *U* test for independent samples for comparing 2 groups and Kruskal Wallis test in comparing more than 2 groups. For comparing categorical data, Chi square (χ^2^) test was performed. Exact test was used instead when the expected frequency is less than 5. *p* values less than 0.05 was considered statistically significant. All statistical calculations were done using computer program SPSS (Statistical Package for the Social Science; SPSS Inc., Chicago, IL, USA) release 15 for Microsoft Windows (2006).

## Results

This study included 48 patients with MF [24 males (50%) and 24 females (50%)] and 45 age and sex-matched controls [18 males (40%) and 27 females (60%)] (P = 0.3). The demographic and clinical data of the included subjects are summarized in Table 1.

**Table 1:** The demographic and clinical characteristics of MF patients (N = 48).

Variable	N(%)
**Age (years)**^**+**^	38.5(13–66)
**Duration (years)** ^**+**^	4(0.5–32)
**Extent (%)** ^**+**^	40(5.0–90)
**Sex**	
Male	24(50.0)
Female	24(50.0)
**Clinical Variant**	
Classic	34(70.8)
Hypopigmented	14(29.2)
**Phototype**	
III	16(33.3)
IV	26(54.2)
V	6(12.5)
**Stage**	
1a	8(16.7)
1b	40(83.3)

^+^ Data presented as median (range), N: number, %: percentage.

### Vitamin D serum status

The serum vitamin D level was significantly lower in patients [median: 13.8 nmol/L (range: 5.3–33.7 nmol/L)] compared to controls [median: 22.7 nmol/L (range: 8.3–90.1 nmol/L)] (*P* < 0.001).

Vitamin D serum levels did not differ significantly according to the demographic and clinical characteristics of MF patients as presented in Table 2.

**Table 2:** Vitamin D levels in MF patients according to the demographic and clinical characteristics.

Variable	Vitamin D level^+^	P value
**Sex**		0.488
Males	14.4(5.3–33.7)	
Females	13.8(7.5–20.1)	
**Clinical variant**		0.427
Classic	13.3(5.3–29.4)	
Hypopigmented	15.1(12.0–33.7)	
**Stage**		0.188
1a	21.3(12.4–33.7)	
1b	13.8(5.3–21.9)	

^+^ Data presented as median (range)

In addition, no significant correlation was found between vitamin D serum level and the extent (*r* = -0.21, *p* = 0.32) or the duration (*r* = 0.04, *p* = 0.87) of MF in the studied patients.

### VDR polymorphisms

#### *Fok*I polymorphism

The genotype frequencies of the *Fok*I polymorphism in patients and controls are presented in Table 3. The frequency of the CC genotype was significantly higher in patients than controls (20.8 vs. 4.4%, *p* = 0.039). In addition, the frequency of the combined C variant genotypes (TC + CC) was significantly higher among patients (75.0%) than among controls (57.7%) (*p* = 0.032).

**Table 3:** The genotype frequencies of *Fok*I polymorphism in the MF patients (N = 48) and (N = 45) controls.

FokI genotype	MF patients N(%)	Controls N(%)	P value
**TT**	12(25.0)	19(42.2)	0.401
**TC**	26(54.2)	24(53.3)	
**CC**	10(20.8)	2(4.4)	0.039
**TC+CC**	36(75.0)	26(57.7)	0.032

N: number, %: percentage

#### *Bsm*I polymorphism

The genotype of *Bsm*I polymorphism in patients and controls is presented in Table 4. Despite no significant difference in genotypic frequencies found between both groups, the frequency of the GG genotype was relatively lower among MF patients than controls (33.3 vs. 37.7%). In addition, the frequency of the combined G variant genotypes (AG + GG) was lower among patients (75%) than controls (84.4%).

**Table 4:** The genotype frequencies of *Bsm*I polymorphism in the MF patients (N = 48) and (N = 45) controls.

BsmI	MF patients N(%)	Controls N(%)	P value
**AA**	12(25.0)	7(15.6)	0.332
**AG**	20(41.7)	21(46.7)	
**GG**	16(33.3)	17(37.7)	
**AG+GG**	36(75.0)	38(84.4)	0.492

N: number, %: percentage

#### *Taq*I polymorphism

The genotype of *Taq*I polymorphism in patients and controls is presented in Table 5. There was no significant difference in the genotypic frequencies among the studied groups. However, the frequency of the TT genotype was relatively lower among patients than controls (29.2 vs. 42.2%). Meanwhile, the frequency of the combined T variant genotypes (CT + TT) was quite higher among patients (75.0%) than controls (71.1%).

**Table 5:** The genotype frequencies of *Taq*I polymorphism in the MF patients (N = 48) and (N = 45) controls.

TaqI genotype	MF patients N(%)	Controls N(%)	P value
**CC**	12(25.0)	13(28.9)	0.610
**CT**	22(45.8)	13(28.9)	
**TT**	14(29.2)	19(42.2)	
**CT+TT**	36(75.0)	32(71.1)	0.360

N: number, %: percentage

Vitamin D serum levels differed significantly with the genotype of *FOK*I polymorphism only, whereas the other studied VDR gene polymorphisms did not show such significant difference (Table 6).

**Table 6:** Vitamin D levels in MF patients according to the different genotypes of VDR gene polymorphisms.

Variable	Vitamin D level^+^	P value
**FokI**		0.014
TT	13.1(5.3–14.2)	
TC	15.1(8.1–33.7)	
CC	18.7(9.2–29.4)	
**TaqI**		0.566
CC	13.1(9.2–21.9)	
CT	15.8(8.1–33.7)	
TT	15.4(12.1–18.0)	
**BsmI**		0.649
AA	13.9(8.1–33.7)	
AG	13.2(5.3–21.9)	
GG	15.4(12.1–20.1)	

^+^ Data presented as median (range)

There was no statistically significant difference in the distribution of the different genotypes of VDR gene polymorphisms among the clinical variants of MF in the studied patients (Table 7).

**Table 7:** Distribution of the genotypes of VDR gene polymorphisms according to the clinical variants (Classic and Hypopigmented) of MF patients.

Variable	Classic	Hypopigmented	P value
**FokI**			0.161
TT	8(26.7%)	2(11.1%)	
TC	16(53.3%)	10(55.6%)	
CC	6(20.0%)	6(33.3%)	
**BsmI**			0.457
AA	8(25.0%)	4(25.0%)	
AG	16(50.0%)	4(25.0%)	
GG	8(25.0%)	8(50.0%)	
**TaqI**			0.134
CC	6(21.4%)	6(30.0%)	
CT	16(57.2%)	6(30.0%)	
TT	6(21.4%)	8(40.0%)	

## Discussion

Vitamin D deficiency has been incriminated in a long list of dermatological diseases [19], and the current study adds MF to this list. This is evident through the significant decrease of the vitamin D level in MF patients in comparison to controls.

This was similar to the findings obtained by a recent study conducted by *Talpur et al*., *2014* [20]. Their study was carried out on 311 CTCL patients, 27 of whom had sezary syndrome (SS), and were compared to 169 cancer controls and 69 normal controls. Their results revealed that vitamin D deficiency is prevalent in CTCL patients with MF or SS. The percentage of CTCL patients (76.9%) who were deficient in vitamin D was similar to other cancer patients (75.2%) as compared to normal healthy control subjects [20].

In the same study, CTCL patients who were deficient in vitamin D were given vitamin D supplements, in an attempt to replenish the stores. Some patients were supplemented with oral vitamin D2 and others were given oral vitamin D3. A difference was found in the correction rate based on the type of vitamin D oral supplement. Patients treated with 1000 IU of oral vitamin D3 were less likely to achieve normal serum levels than those treated with 50,000 IU of vitamin D2 twice a week. This difference can be accounted for by the higher cumulative dose received by the vitamin D2 group or by patient compliance [20], as the authors reported.

Taking a step further, the current study was concerned with the gene polymorphism of the VDR being the most widely studied genetic factor implicated in vitamin D deficiency [21]. Furthermore, there is a presumption that prolonged antigen stimulation leads to the emergence of aberrant clones of T cells that are deficient in Fas-mediated apoptosis and secrete Th2 cytokines. Those incriminated cells were found to express VDR [22], adding more to the importance of studying the VDR polymorphism in MF cases. To our knowledge no previous studies tackled this point of research in the context of MF.

The three chosen SNPs (*Fok*l, *Bsm*l, or *Taq*l) of the VDR are among the most common to be found incriminated in various diseases and malignancies [12]. The current work detected a significant difference of *Fok*I polymorphism frequencies (both the CC and the combined C variant genotypes) among MF patients compared to controls. On the other hand, such significant difference could not be detected with the other studied VDR polymorphisms (*Bsm*l and *Taq*l).This finding sheds the light on the potential involvement of *Fok*I polymorphism in MF and its possible interaction with VDR polymorphisms other than those assessed (*Bsm*l and *Taq*l) in the present study. In other words, *Fok*I polymorphism may be in linkage disequilibrium with other functionally relevant variants [23, 24]. Also, the relatively limited number of recruited patients and controls could account for the lack of significant difference in the patterns of expression of *Bsm*l and *Taq*l polymorphisms. Accordingly, larger scale studies are recommended to verify the exact role of the studied genes.

The role played by vitamin D deficiency in the pathogenesis of MF might be explained by several pathways. Primarily, vitamin D promotes paracrine activation of T- and B- lymphocytes, and decreases production of Th1 cytokines [25]. In addition, the cathelicidin gene is regulated by vitamin D/VDR [25] and vitamin D encodes antimicrobial peptides [26–29]; as defensin. As part of the innate immune system, defensin protects skin against pathogenic organisms [28]. Hence, vitamin D deficiency can lead to increased colonization by staphylococcus aureus and sepsis that is common in patients with CTCL [28]. Staphylococcal super-antigen can cause persistent antigen stimulation and promote T-cell activation, therefore initiating the rise of incriminated aberrant clones [30].

It is also worth noting that MF lesions usually first appear on sun-shielded skin and improve with phototherapy. Whether phototherapy treats MF by raising local and/or systemic vitamin D levels is an area of research to be explored [20]. All our patients were known to have received phototherapy (by history taking) prior to their inclusion in the study (as all patients were instructed to refrain from any kind of treatment 4 weeks before their enrollment). Logically, there was no significant difference in vitamin D level among them. Thus, to evaluate the real impact of phototherapy on vitamin D levels, studies measuring serum vitamin D in MF patients receiving phototherapy versus other lines of treatment should be conducted.

Vitamin D is unfolding as a promising therapeutic option for management of cancer and autoimmunity [31]. Supplementation with vitamin D goes hand in hand with chemotherapeutic agents such as doxorubicin in treatment of advanced-stage CTCL patients [32]. Several studies have reported inverse relationships between serum 25-hydroxyvitamin D (25[OH] D3) levels and incidence or mortality of adenocarcinomas in the colon, breast, ovary, lung, endometrium, kidney, pancreas, non-Hodgkin lymphoma (NHL), and multiple myeloma [25]. Increased sun exposure, which can be used as an indirect marker of long-term vitamin D status, was associated with less risk of NHL [33], including B-cell lymphoma [34]. Poorer outcomes in NHLs were associated with low vitamin D levels in a large study performed on patients with lymphoma [35]. However, studies to determine the relationship between vitamin D status and MF in particular were scarce in literature.

There was no statistically significant correlation found between vitamin D serum level and the extent, clinical variants or duration of MF. This could be explained by the notion that vitamin D might be involved in the occurrence of MF -as a malignant disease- rather than having a role in determining the clinical variants or the duration and extent of MF. Moreover, the lack of such a relationship might be attributed to the multi-factorial nature of the disease [36]. Nonetheless, the absence of a significant correlation does not absolutely abolish the important role played by vitamin D in MF.

The distribution of the various genotypes of VDR gene polymorphisms did not show a significant difference among the clinical variants of MF patients. In contrast, there was a significant difference in vitamin D level in MF patients with *Fok*I polymorphism, pointing again to the importance of this SNP in the pathogenesis of MF.

In conclusion, decreased vitamin D serum level and *Fok*I polymorphism seem to comprise a role in the context of MF, according to the results of the present study. VDR gene polymorphisms warrant further larger scale investigations to detect the exact genes involved in the pathogenesis of such an enigmatic disease.

## Supporting Information

S1 Table Master Table for Demographic and Clinical Data of Patients and Controls. (XLSX)
